# Anakinra reduces lung inflammation in experimental acute lung injury

**DOI:** 10.1002/iid3.548

**Published:** 2021-12-09

**Authors:** Paul Engeroff, Aude Belbézier, Antoine Monsel, David Klatzmann

**Affiliations:** ^1^ INSERM, Immunology‐Immunopathology‐Immunotherapy Department (i3) Sorbonne Université Paris France; ^2^ Multidisciplinary Intensive Care Unit, Department of Anesthesiology and Critical Care AP‐HP, Hôpital Pitié‐Salpêtrière Paris France; ^3^ Biotherapy (CIC‐BTi) and Inflammation‐Immunopathology‐Biotherapy Department (i2B) AP‐HP, Hôpital Pitié‐Salpêtrière Paris France

**Keywords:** covid‐19, IL‐1, IL‐1Ra, immunotherapy, respiratory distress syndrome

## Abstract

**Introduction:**

Acute respiratory distress syndrome (ARDS) is a severe form of acute lung injury (ALI) resulting in life‐threatening hypoxaemia. Although ARDS can be caused by a variety of pathogens or major trauma, it is best known as the major cause of mortality in COVID‐19 patients. Since ARDS is often associated with dysregulated inflammatory immune responses, immunomodulatory approaches represent a possible treatment option. The objective of this study was to evaluate the therapeutic potential of interleukin (IL)‐1 blockade using Anakinra in a mouse model of lipopolysaccharide (LPS)‐induced ALI.

**Methods:**

We evaluated the effects of a daily subcutaneous Anakinra treatment in a mouse model of LPS‐induced ALI. We monitored body weight to assess the general health status of the mice. Two days after ALI induction, we evaluated the inflammatory cytokine MIP‐2 as well as protein levels in bronchoalveolar lavage (BAL) fluids. Two and nine days after ALI induction, we evaluated infiltrating leukocytes in BAL fluid and lung tissue.

**Results:**

Anakinra treatment reduced ALI‐induced weight loss compared to nontreated groups. At Day 2, Anakinra treatment reduced levels of MIP‐2 and protein in BAL fluids and reduced frequencies of NK cells and neutrophils in the lung tissue. Nine days after ALI induction, Anakinra treated mice displayed reduced levels of neutrophils and alveolar macrophages in BAL fluids.

**Conclusions:**

IL‐1 blockade using Anakinra reduced classical hallmarks of inflammation in a mouse model of ALI. Our data support ongoing and future research on the evaluation of Anakinra as a potential treatment option in ARDS.

## Graphical abstract

Acute respiratory distress syndrome (ARDS) is a severe lung disease currently lacking specific therapeutics. We show that interleukin‐1 blockade with Anakinra improves an experimental animal model of ARDS.

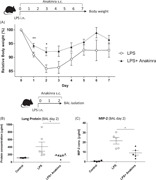

## 1 INTRODUCTION

Acute respiratory distress syndrome (ARDS) is characterized by an acute onset of hypoxaemia, reduced lung compliance, inflammatory (noncardiogenic) pulmonary oedema and requirement of mechanical ventilation. ARDS is most often caused by bacterial or viral pneumonia, nonpulmonary sepsis or major trauma. Even before the COVID‐19 pandemic, up to 10% of patients admitted to the intensive care units and 25% of mechanically ventilated patients displayed features of ARDS.^1^, ^2^ Recently, ARDS has gained worldwide attention as the leading cause of severe cases and mortality in COVID‐19.^3^ ARDS therapy, including in COVID‐19, primarily relies on supportive treatments such as protective ventilation, neuromuscular blocking agents, and prone positioning while mortality of the syndrome remains high, nearing 30%.^1^

“Hyper” and “hypo‐inflammatory” phenotypes of ARDS have been distinguished on the basis of expression levels of circulating proinflammatory cytokines and chemokines,^4^ among which interleukin (IL)‐1 that has been shown to be involved in the immunopathology of acute lung injury (ALI).^5^, ^6^, ^7^ In COVID‐19, the exact phenotype is still a matter of debate, but proinflammatory cytokines were shown to be dysregulated.^3^, ^8^ A recent study showed that Anakinra (Kineret®), a recombinant version of the IL‐1 receptor antagonist IL‐1Ra, is beneficial in patients with severe forms of COVID‐19.^9^ Additional studies have come out since and shown positive trends regarding efficacy and safety of Anakinra in COVID‐19.^10^, ^11^ Here, we show that Anakinra improves an experimental mouse model of ALI that is commonly used to study ARDS^12^ by reducing (i) lung inflammation, (ii) lung permeability for proteins, and (iii) neutrophil, NK cell and alveolar macrophages influx into the alveoli.^12^, ^13^ These findings support research in the optimisation of Anakinra‐based therapy in the acute phase of ARDS with dysregulated inflammation.

## 2 METHODS

### 2.1 Mouse model

C57BL/6 mice (Charles River) were kept at the UPMC Centre d'Expérimentation Fonctionnelle animal facility (Paris, France). All protocols were approved by the local ethics committee and according with the European legislation on animal care, housing, and scientific experimentation. Lipopolysaccharide (LPS) from *Escherichia coli* O55:B5 (Sigma Aldrich) was applied by intranasal instillation at 10 mg/kg. Anakinra (Kineret®, Swedish Orphan Biovitrum) was given at 50 mg/kg subcutaneously simultaneously with twice per day. Mice were either treated only with LPS or with LPS + Anakinra. Body weight was assessed daily for 7 days. At either Day 2 or Day 9, bronchoalveolar lavage (BAL) fluid and lung tissue were isolated. Lung tissue was thinly cut using scissors and digested with 0.3 PZ units Liberase TN (Sigma Aldrich) and 0.2 mg/ml DNAse (Sigma Aldrich) in complete RPMI 1640 medium (Sigma Aldrich) for 20 min at 37°C. The cells were further purified using 80% Percoll (Sigma) gradients on which cells were layered with 40% Percoll. Lung protein and MIP‐2 levels were assessed from BAL fluid using Pierce Micro BCA Protein Assay Kit (Thermo Fisher Scientific) and a mouse MIP‐2 (CXCL2) ELISA kit (Thermo Fisher Scientific), respectively.

### 2.2 Flow cytometry

Immune cells from BAL fluid or lung tissue cells were stained for 20 min at 4°C with Biotin rat anti‐mouse CD45 (clone 30‐F11; BD). Thereafter, cells were labelled for 20 min at 4°C with APC‐A750 Streptavidin (BD), APC rat anti‐mouse CD11c (clone N418; Thermo Fisher Scientific), APC‐A700 rat anti‐mouse CD11b (clone M1/70, Thermo Fisher Scientific), FITC rat anti‐mouse Ly6G (clone RB6‐8C5; Thermo Fisher Scientific), PE rat anti‐mouse Siglec F (clone E50‐2440, BD), PE‐CF594 rat anti‐mouse CD3 (clone 145‐2C11; BD), CD19 (clone 1D3, BD) and V500 IA/IE (clone M5/114.15.2; BD). The gating strategy is depicted in Figure S1.

### 2.3 Statistics

**Figure 1:**
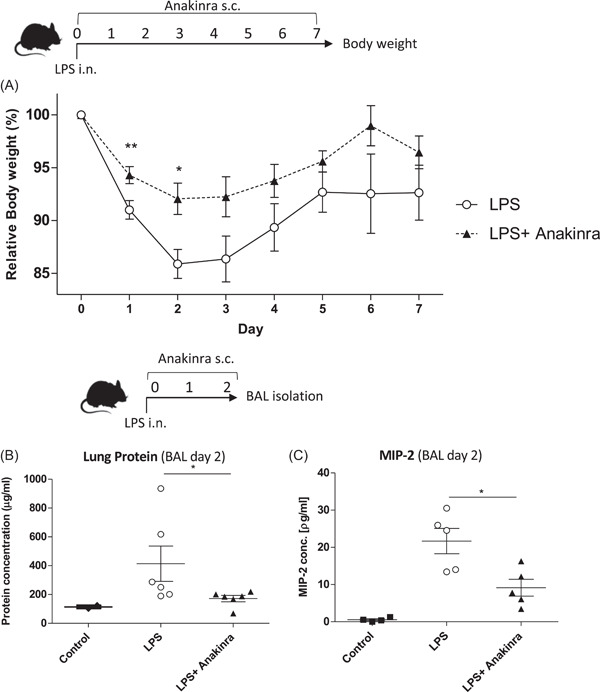
(A) Shown are relative changes in body weight over 7 days in LPS challenged groups (LPS), and LPS challenged + Anakinra‐treated groups (Anakinra) from three independent experiments, *n* = 9. (B) Protein concentration (mean ± *SEM*) in BAL fluids 2 days after LPS challenge assessed by BCA assay from LPS challenged groups (LPS), LPS challenged + Anakinra‐treated groups (LPS + Anakinra) compared to naïve mice (Control). Two independent experiments, *n* = 6. (C) MIP‐2 concentration (mean ± *SEM*) in BAL fluids 2 days after LPS challenge assessed by ELISA, two independent experiments *n* = 5. BAL, bronchoalveolar lavage; ELISA, enzyme‐linked immunoassay; LPS, lipopolysaccharide

Three mice per LPS and LPS + Anakinra groups were used in all experiments. For body weight experiments, nine mice per group from three individual experiments are shown. Body weight experiments include three male mice in LPS and LPS + Anakinra groups. All other mice used for body weight, BAL and lung tissue experiments were females. For BAL and lung tissue cell populations, shown are six mice from two individual experiments where all populations are from the same two experiments. Results are representative for three independent experiments. Naïve mice (controls) are from two individual experiments, using two and one control mouse respectively. BAL lung protein and MIP‐2 levels are from two independent experiments. Statistical tests were performed with GraphPad Prism 6.0 software (GraphPad Software). Figure 1A was analysed by two‐way analysis of variance. All other groups were analysed using two‐tailed Mann–Whitney test (*α* = .05) (*p ≤ .05, **p ≤ .01).

## 3 RESULTS

To monitor the general health status of the mice, we first investigated changes in body weight of LPS only and LPS + Anakinra treated mice with ALI. Mice were monitored over 7 days after LPS challenge. As shown in Figure 1A, Anakinra significantly reduced ALI‐induced weight loss compared to LPS only treated controls, most prominently at Days 2 and 3. At Day 2, Anakinra treatment also led to a significant reduction of BAL protein levels (Figure 1B) as well as MIP‐2 concentration (Figure 1C), which are two classical ALI markers.

**Figure 2:**
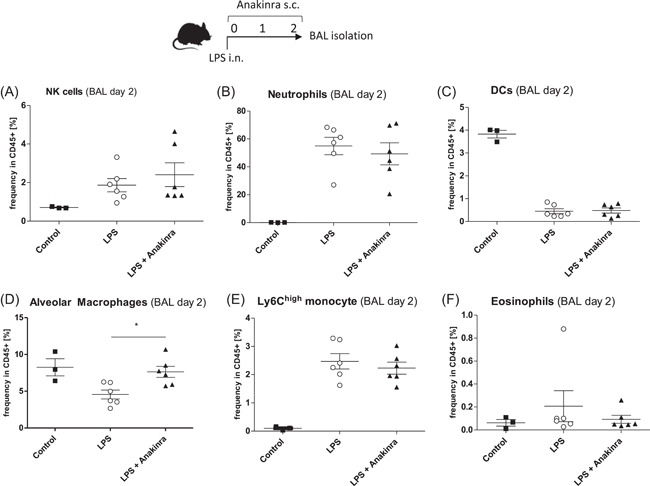
Mice were challenged with LPS (LPS) or additionally treated with Anakinra (LPS + Anakinra) for 2 days before BAL fluid isolation. Shown are mean ± *SEM* cell frequencies in CD45 + cells as assessed by flow cytometry. The data shown are from two independent experiments. (A) Frequency of NK cells (B) Frequency of neutrophils (C) Frequency of dendritic cells (D) Frequency of alveolar macrophages (E) Frequency of Ly6C^hi^ monocytes and (F) Frequency of eosinophils. BAL, bronchoalveolar lavage; LPS, lipopolysaccharide

**Figure 3:**
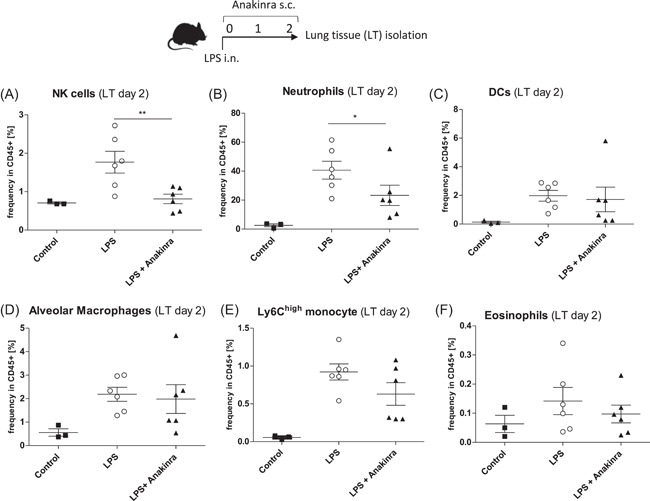
Mice were challenged with LPS (LPS) or additionally treated with Anakinra (LPS + Anakinra) for 2 days before lung tissue isolation. Shown are mean ± *SEM* cell frequencies in CD45+ cells as assessed by flow cytometry. The data shown are from two independent experiments. (A) Frequency of NK cells (B) Frequency of neutrophils (C) Frequency of dendritic cells (D) Frequency of alveolar macrophages (E) Frequency of Ly6C^hi^ monocytes and (F) Frequency of eosinophils. LPS, lipopolysaccharide

We then aimed to investigate leukocyte infiltration into the lung. In a first step, we investigated early cellular inflammation at Day 2 in BAL fluids (Figure 2). Although our model showed strong neutrophil infiltration, a hallmark of ALI, we did not observe differences in BAL fluid neutrophils between LPS only and LPS + Anakinra groups (Figure 2B). The same was also true for other cell populations beside an increase in alveolar macrophages in Anakinra treated mice compared to LPS only treated mice (Figure 2D). In a next step, we evaluated immune cells in the lung tissue. Interestingly, we observed a striking reduction in lung NK cells (Figure 3A) as well as a reduction in neutrophils (Figure 3B). In contrast, frequencies of lung DCs, alveolar macrophages, Ly6C^hi^ mononcytes, and eosinophils did not change significantly (Figure 3C–F).

**Figure 4:**
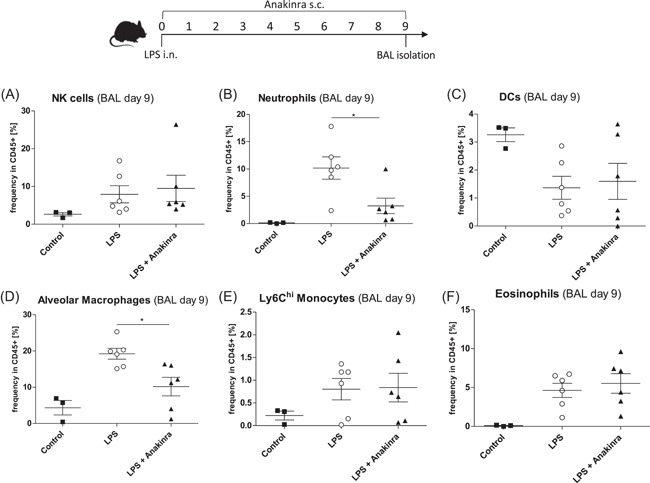
Mice were challenged with LPS (LPS) or additionally treated with Anakinra (LPS + Anakinra) for 9 days before BAL fluid isolation. Shown are mean ± *SEM* cell frequencies in CD45+ cells as assessed by flow cytometry. The data shown are from two independent experiments. (A) Frequency of NK cells (B) Frequency of neutrophils (C) Frequency of dendritic cells (D) Frequency of alveolar macrophages (E) Frequency of Ly6C^hi^ monocytes and (F) Frequency of eosinophils. LPS, lipopolysaccharide

To examine the effects of Anakinra on later‐stage cellular inflammation, we evaluated the cell populations at Day 9 post LPS challenge. At this time point, BAL fluid and lung tissue showed similar results (not shown), we therefore proceeded to measure BAL only. In Anakinra‐treated mice, Neutrophils were reduced at Day 9 (Figure 4B). Anakinra also led to a reduction in BAL alveolar macrophages (Figure 4D) while DCs, NK cells, Ly6C^hi^ monocytes, and eosinophils were not different at this time point (Figure 4A, 4C, 4E, 4F).

## 4 DISCUSSION

In our model of LPS‐induced ALI, the observed cellular kinetics, with an early recruitment of neutrophils and a later recruitment of macrophages as well as higher levels of pulmonary MIP‐2 and protein levels is consistent with previous studies.^2^, ^12^, ^13^ Anakinra mitigated the key markers of local inflammation and restored lung protein permeability. Those results are consistent with previous reports that have demonstrated a key role for IL‐1 in ALI.^5^, ^6^, ^14^ Reduced neutrophil infiltration specifically is in line with the known role of IL‐1 in neutrophil recruitment in ALI.^5^ Interestingly, Anakinra also had a substantial effect on NK cells. Previous studies have demonstrated an involvement of NK cell in neutrophil recruitment during ALI.^15^, ^16^ Interestingly, NK cells have recently also gained attention in COVID‐19.^17^ A role for IL‐1 in NK cell activation is plausible as it was previously reported that IL‐1 can regulate IFN‐γ production from NK cells.^18^ At Day 2, we observe a higher frequency of alveolar macrophages in BAL fluids of Anakinra‐treated groups. Potentially, those cells could contribute to the protective effect of Anakinra. An interesting report has shown that macrophages can produce IL‐1Ra to attenuate inflammation.^19^ Hence, it could be hypothesized that IL‐1 promotes NK cell and neutrophil infiltration while macrophages suppress inflammation. To what extent Il‐1 blockade with Anakinra has a prominent effect on one specific cell type or whether it affects multiple cell types in more general fashion remains to be elucidated in further detail. In our hands, subcutaneous treatment was required to observe the most beneficial effects, likely due to the relatively short half‐life of Anakinra. An anti‐inflammatory effect of intratracheal Anakinra was observed in a previous study.^14^ However, no differences in cellular inflammation were found, which is in line with our results regarding changes in BAL neutrophils. However, in our study, there is a clear effect on NK cells and neutrophils in the lung tissue. The differences between BAL and lung tissue in the early phase of ALI are an interesting aspect of our findings and suggest that in the early phase, the protective effect of Anakinra may take place in the tissue.

Hence, all our results regarding Anakinra reducing lung inflammation are in line with previous reports and support its further evaluation to optimize treatment modalities and efficacy in ARDS and potentially in COVID‐19 patients.

## FUNDING

Assistance Publique‐Hôpitaux de Paris, Investissements d'Avenir programme (ANR‐11‐IDEX‐0004‐02, LabEx Transimmunom) and Swiss National Science Foundation (SNF grant P2BEP3_188262 to Paul Engeroff).

## CONFLICT OF INTERESTS

The authors declare that there are no conflict of interests.

## AUTHOR CONTRIBUTIONS

**Paul Engeroff** and **Aude Belbézier**: performed experiments and wrote the manuscript. **Antoine Monsel**: designed and interpreted the results. **David Klatzmann**: designed, interpreted, and supervised the study.

## Supporting information


**Suppl. Figure 1** In CD45+ cells, alveolar macrophages and dendritic cells were separated as CD11c+MHCII+ cells whereas alveolar macrophages were then distinguished from dendritic cells by Siglec F expression. Thereafter, B and T cells as well as CD11b‐ cells were excluded and neutrophils were gated as Ly6G+ cells. In all Ly6G‐ cells, NK cells were defined as NK1.1+, whereas Ly6C^hi^ monocytes were defined as Ly6C+.
