# Supplementary figures and images for: Anakinra reduces lung inflammation in experimental acute lung injury
Source: Immun Inflamm Dis. 2021 Dec 9;10(2):123–9. doi: 10.1002/iid3.548 (PMC8767508; doi:10.1002/iid3.548)

Suppl. Figure 1

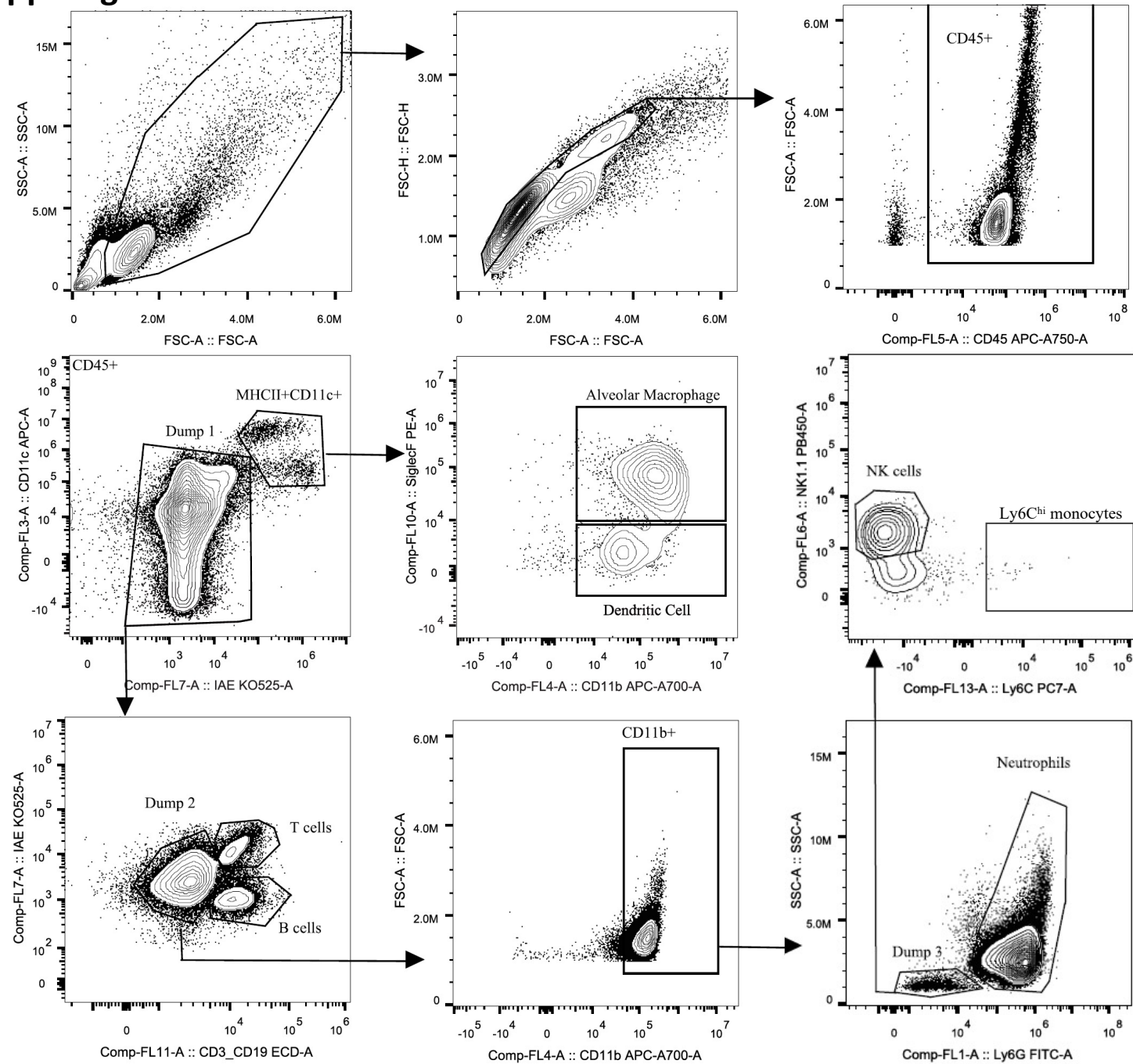

Supplement: Supplementary file 1 — Suppl. Figure 1 In CD45+ cells, alveolar macrophages and dendritic cells were separated as CD11c+MHCII+ cells whereas alveolar macrophages were then distinguished from dendritic cells by Siglec F expression. Thereafter, B and T cells as well as CD11b‐ cells were excluded and neutrophils were gated as Ly6G+ cells. In all Ly6G‐ cells, NK cells were defined as NK1.1+, whereas Ly6Chi monocytes were defined as Ly6C+. [file IID3-10-123-s001.pdf]
